# Utilization of Fishery Processing By-Product Squid Pens for α-Glucosidase Inhibitors Production by *Paenibacillus* sp.

**DOI:** 10.3390/md15090274

**Published:** 2017-08-30

**Authors:** Van Bon Nguyen, Anh Dzung Nguyen, San-Lang Wang

**Affiliations:** 1 Department of Science and Technology, Tay Nguyen University, Buon Ma Thuot 630000, Vietnam; bondhtn@gmail.com; 2 Institute of Biotechnology and Environment, Tay Nguyen University, Buon Ma Thuot 630000, Vietnam; nadzungtaynguyenuni@yahoo.com.vn; 3 Department of Chemistry, Tamkang University, New Taipei City 25137, Taiwan; 4 Life Science Development Center, Tamkang University, New Taipei City 25137, Taiwan

**Keywords:** squid pens, α-glucosidase inhibitor, *Paenibacillus* species, diabetes, obesity, microbial conversion, fishery processing, homogentisic acid

## Abstract

The supernatants (the solution part received after centrifugation) of squid pens fermented by four species of *Paenibacillus* showed potent inhibitory activity against α-glucosidases derived from yeast (79–98%) and rats (76–83%). The inhibition of acarbose—a commercial antidiabetic drug, used against yeast and rat α-glucosidases—was tested for comparison; it showed inhibitory activity of 64% and 88%, respectively. Other chitinolytic or proteolytic enzyme-producing bacterial strains were also used to ferment squid pens, but no inhibition activity was detected from the supernatants. *Paenibacillus* sp. TKU042, the most active α-glucosidase inhibitor (aGI)-producing strain, was selected to determine the optimal cultivation parameters. This bacterium achieved the highest aGI productivity (527 µg/mL) when 1% squid pens were used as the sole carbon/nitrogen source with a medium volume of 130 mL (initial pH 6.85) in a 250 mL flask (48% of air head space), at 30 °C for 3–4 d. The aGI productivity increased 3.1-fold after optimization of the culture conditions. Some valuable characteristics of *Paenibacillus* aGIs were also studied, including pH and thermal stability and specific inhibitory activity. These microbial aGIs showed efficient inhibition against α-glucosidases from rat, yeast, and bacteria, but weak inhibition against rice α-glucosidase with IC_50_ values of 362, 252, 189, and 773 µg/mL, respectively. In particular, these aGIs showed highly stable activity over a large pH (2–13) and temperature range (40–100 °C). Various techniques, including: Diaoin, Octadecylsilane opened columns, and preparative HPLC coupled with testing bioactivity resulted in isolating a main active compound; this major inhibitor was identified as homogentisic acid (HGA). Notably, HGA was confirmed as a new inhibitor, a non-sugar-based aGI, and as possessing stronger activity than acarbose with IC_50,_ and maximum inhibition values of 220 μg/mL, 95%, and 1510 μg/mL, 65%, respectively. These results suggest that squid pens, an abundant and low-cost fishery processing by-product, constitute a viable source for the production of antidiabetic materials via fermentation by strains of *Paenibacillus*. This fermented product shows promising applications in diabetes or diabetes related to obesity treatment due to their stability, potent bioactivity, and efficient inhibition against mammalian enzymes.

## 1. Introduction

Squid pens (SP), a chitinous marine material obtained as by-products from seafood processing, have been used to produce various bioactive materials via microbial conversion. SPs has been extensively studied for its use in the biosynthesis of chitooligomers [1], antioxidants [2], exopolysaccharides [3,4,5], some enzymes [1,6], and biosorbents [7,8]. SP has been also found to be the richest source of chitin and chitosan, biomaterials reported as having many potential applications [6]. After discovering its newly beneficial applications in drugs, SP was used in this study for conversion to active antidiabetic materials.

Diabetes mellitus (DM) is recognized as a continuing and growing health issue, seriously reducing the quality of people’s health worldwide [9]. This disease has also become a major cause of death, responsible for 8.4% of total deaths in 2013 [10]. While DM falls into two types, around 90% of DM cases are type 2 [11]. Several therapies for type 2 diabetes exist, including α-glucosidase inhibitors (aGIs) [12].

aGIs may be obtained from medicinal herbs or chemical synthesis [13]. However, it has been reported that aGIs are difficult to obtain in large amounts from herbs [14], while synthesized aGIs, although able to be produced on a large scale, often cause side effects [15]. Microbial conversion may offer another means of obtaining natural aGIs with promising activity, providing an alternative to commercially available inhibitors—such as acarbose, miglitol, and voglibose—which have been reported to cause side effects [16].

Several novel aGI-producing microbes have been studied. Of these, genus *bacillus* [17,18,19,20,21], *Streptomyces* [22,23], *Actinoplanes* spp. SE-50 [24], and *Stenotrophomonas maltrophilia* [25] have been extensively explored for aGI production, with the use of soybeans as the major carbon/nitrogen (C/N) source. In our previous study, *Paenibacillus* sp. TKU042 was found to be an active aGI-producing strain [16]; in fact, this bacterium was determined to be the most potent aGI-producing strain among more than 600 bacterial strains isolated from the soils of Northern Taiwan. Nutrient broth fermented by these bacteria demonstrated acarbose-comparable activity in our previous report [16]. In this study, an effort was made to use fishery by-products for efficient aGI production via microbial conversion. Four novel species of *Paenibacillus* and some chitinolytic and/or proteolytic enzyme–producing strains were used for the fermentation of squid pens powder (SPP). Optimal time and parameters for the enhancement of aGI productivity were explored. The specific inhibitory activity, stability of the aGIs, and the major active compound were also investigated.

## 2. Results and Discussion

### 2.1. Production of aGIs from SPP by Paenibacillus and Some Chitinolytic and/or Proteolytic Enzyme-Producing Bacterial Strains

*Paenibacillus* sp. TKU042 induced active aGIs in our previous study [16]. To investigate the potency of aGI production by the genus *Paenibacillus*, four novel species of *Paenibacillus* were cultivated in a medium containing 1% SPP before the culture supernatants of fermented SPP (FSPP) were tested for activity. The results in Table 1 show that all tested species of *Paenibacillus* exhibit the same manner of aGI production. Supernatants of fermented cultures from these species inhibited α-glucosidases from both rats and yeast, with maximum activity of 76–83% and 79–98%, respectively, while productivity was in the range of 298–335 U/mL and 450–560 U/mL, respectively. FSPPs showed comparable or higher maximum inhibition compared to acarbose against yeast and rat α-glucosidases (88% and 64%, respectively). *Paenibacillus* species are valuable due to their novel and potent applications in medicine, industry, health food, and agriculture [16]. These bacteria have been reported as producing enzymes [26,27,28,29], biological control agents [29,30], biosurfactants [5], biofertilizers [31,32], antioxidants [2], and exopolysaccharides [3,5]. For this study, the function of *Paenibacillus* species as aGI producers was investigated based on recent literature reviews.

In many reports, soybeans have been used as the major C/N source for aGI production via microbial conversion [17,18,22,33,34]. Nutrient broth was also used as the sole C/N source for the biosynthesis of aGIs [16]. Unlike previous reports, an abundant and low-cost material obtained from fishery by-products, SPP, was used in this study for aGI production via conversion by *Paenibacillus*. 

SPP was the sole C/N source in this experiment; it contains approximately 40% chitin and 60% protein [35]. As such, we wondered if the chitinolytic or proteolytic enzyme-producing bacterial strains could have the same function in aGI production from SPP as *Paenibacillus*. Therefore, four bacteria producing chitinolytic or proteolytic enzymes were also used to ferment the SPP. Unexpectedly, the culture supernatants of SPP fermented by these strains showed no inhibitory activity (Table 1). However, this result confirms that the aGIs were produced via bioconversion of SPP by *Paenibacillus*, and did not exist in the material beforehand. *Paenibacillus* sp. TKU042, the most active strain, was chosen for further investigation.

### 2.2. Optimization of the Fermentation Process for Maximal aGI Productivity

#### 2.2.1. Effects of Cultivation Time and Supplementary Air on aGI Productivity

**Figure 1:**
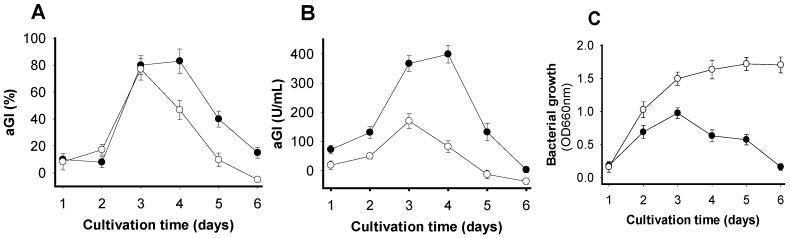
The effects of time and supplementary air on aGI production via fermentation with *Paenibacillus* sp. TKU042, using SPP as the sole C/N source. (-●-): no supplementary air; (-o-): supplementary air once per day. (**A**) aGI activity expressed as %; (**B**) aGI activity expressed as U/mL; (**C**) bacteria growth expressed as OD660.

To determine the optimal time for obtaining the highest aGI productivity, SPP was fermented by *Paenibacillus* sp. TKU042 for 6 d. Fermentation was performed in a 250 mL Erlenmeyer flask with 100 mL of medium containing 1% SPP concurrently under two different sets of conditions (no supplementary air, and supplementary air once per day). The culture supernatants were harvested daily and used to detect activity and bacterial growth. As shown in Figure 1A, the supernatants of FSPP showed maximum activity (≥80%) under both sets of conditions on day 3. After that, activity dramatically decreased in the population given supplementary air during cultivation, but still increased slightly in the culture without supplementary air up to day 4. To clarify which cultivation conditions would give higher aGI productivity, the supernatants were appropriately diluted to obtain inhibitory activity (around 50%), then expressed as U/mL. The notable difference in aGI productivity between the two sets of conditions is clearly observed in Figure 1B. The results indicated that cultivation without supplementary air could result in much higher aGI productivity (approximately two- to three-fold higher on day 3 and day 4, respectively) than if supplementary air was provided. Thus, the cultivation with no supplementary air was chosen for subsequent experiments, and a cultivation time of 3–4 d was considered suitable to harvest active aGIs. 

Bacterial growth was also recorded, as illustrated in Figure 1C. The relationship between aGI productivity and bacterial growth when cultivated with supplementary air was clearly observed from day 1 to day 3. The enhancement of aGI is similar to that of bacterial growth. Similarly, in a culture without supplementary air, this same relationship was observed from day 1 to day 4. However, after day 4 of cultivation, bacterial growth continued to slightly increase, while aGI productivity dramatically decreased.

#### 2.2.2. Effects of Some Parameters on aGI Productivity

**Figure 2:**
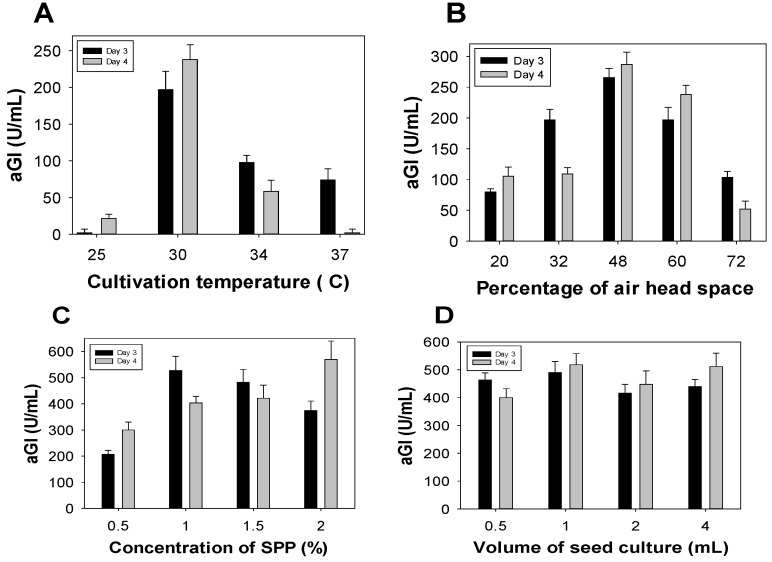
Effects of specific parameters on aGI productivity, including: cultivation temperature (**A**); percentage of air head space (**B**); concentration of SPP (**C**); and volume of bacterial seed culture (**D**).

To achieve greater aGI productivity, some fermentation parameters were investigated for their effects, including: cultivation temperature, percentage of air head space in the 250 mL-Erlenmeyer flask, concentration of SPP, and amount of bacterial seed. As shown in Figure 2A, *Paenibacillus* sp. TKU042 demonstrated the greatest aGI productivity (240 U/mL, day 4) at 30 °C, with no or low productivity (0–98 U/mL) at other culture temperatures (25 °C, 34 °C, and 37 °C). Therefore, 30 °C was used to determine the optimal percentage of air head space; the results are presented in Figure 2B. Cultivation in 48% of air head space resulted in the greatest productivity (287 U/mL), and was therefore selected for the subsequent experiment exploring the effects of SPP concentration. 

The results (Figure 2C) show that aGI synthesis reached its highest productivity at SPP concentrations of 1–2%. Taking material costs into account, 1% SPP was ultimately chosen for the subsequent tests. The amount of bacterial seed was also investigated for its effect on aGI productivity, using the optimal parameters established above. However, this factor had no effect (Figure 2D).

In summary, aGIs were efficiently synthesized by *Paenibacillus* sp. TKU042 under optimal cultivation conditions in a 250 mL Erlenmeyer flask with 130 mL of medium (initial pH 6.85) containing 1% SPP, 0.1% K_2_HPO_4_, and 0.05% MgSO_4_·7H_2_O. Fermentation was performed in an incubator at 30 °C, a shaking speed of 150 rpm, and no supplementary air for 3 d. aGI productivity was increased 3.1-fold after optimization.

### 2.3. Specific Inhibition of FSPP

Six sources of commercial enzymes were tested to explore the potent specific inhibition of *Paenibacillus* sp. aGIs for development as an antidiabetic drug. The inhibitory activity was calculated and expressed as IC_50_ (µg/mL) and maximum inhibition (%) values. The concentration of an inhibitor that could inhibit 50% activity of an enzyme was defined as IC_50_ value; therefore, the lower this value, the stronger the inhibitor. As shown in Table 2, FSPP demonstrated potent inhibition against yeast α-glucosidase (252 µg/mL and 99%), rat α-glucosidase (362 µg/mL and 82%) and bacterial α-glucosidase (189 µg/mL and 85%). However, it showed weaker inhibition against α-glucosidase from rice (773 µg/mL and 60%), and showed no inhibition against porcine pancreatic and *B. subtilis* α-amylases.

α-glucosidase from yeast has been reported to be widely used to evaluate aGI activity; however, rat α-glucosidase was found to be a more valuable source for this purpose [16]. Although FSPP showed weaker inhibitory activity against bacterial and rice α-glucosidases, it demonstrated inhibition comparable to, or much stronger than, acarbose against rat and yeast α-glucosidases; therefore, FSPP could be a potential α-glucosidase inhibitor for possible treatment for diabetes or obesity-related diabetes. 

In our previous study [16], nutrient broth fermented by *Paenibacillus* sp. TKU042 (FNB) had the same specific inhibitory activity as FSPP. FNB also showed efficient inhibition against yeast, rat, and bacterial α-glucosidases, but weaker inhibition against rice α-glucosidase. No inhibition against α-amylases was observed; thus, FSPP and FNB demonstrate similarities in specific activity. 

### 2.4. The pH and Thermal Stability of FSPP

**Figure 3:**
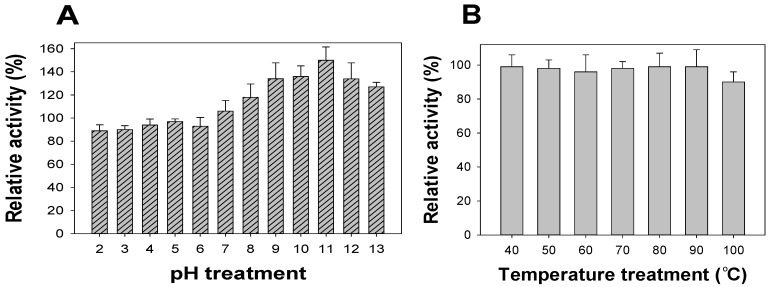
The pH and thermal stability of FSPP. The pH (**A**) and thermal (**B**) stabilities of FSPP were determined by treating FSPP in the pH range of 2–13 and temperature range of 40–100 °C for 30 min, respectively; aGI activity was then tested under the same conditions using the enzymatic inhibition assay mentioned in the methods section.

pH stability has been suggested as an important characteristic for evaluating aGIs. A potent inhibitor should be stable in acidic conditions since the pH in the gastrointestinal tract is normally very acidic [16,36,37]. To determine its pH stability, FSPP was pre-treated for 30 min at pH 1–13 before the inhibition was tested at pH 7, using the enzymatic inhibition assay mentioned in the methods section; the results are illustrated in Figure 3A. FSPP was very stable from pH 2–7, with high relative activity of 90–106%. From pH 8–13, FSPP increased its inhibition with a greater relative activity of 118–150%. The thermal stability of FSPP was also investigated by pre-incubating the sample at a high temperature (40–100 °C) for 30 min before activity was measured at 37 °C using the same inhibition assay described above. As shown in Figure 3B, FSPP also demonstrated great thermal stability with relative activity of approximately 100% in the temperature range of 40–90 °C. aGI activity could remain at 90% even if FSPP was treated at 100 °C for 30 min.

aGIs from *Euonymus laxiflorus* Champ methanolic extract (ELC) and fermented nutrient broth (FNB) were reported to be thermally stable at 100 °C (heated for 30 min) with relative activities of 90% [36] and 92% [16], respectively. As such, they have the same thermal stability as FSPP; however, FSPP aGIs demonstrated slightly higher, or even much greater, pH stability than those of FNB and ELC, with relative activities of 90–95%, 80–93% and 48–52%, respectively. 

### 2.5. Isolation and Identification of the Major Inhibitor from FSPP

**Figure 4:**
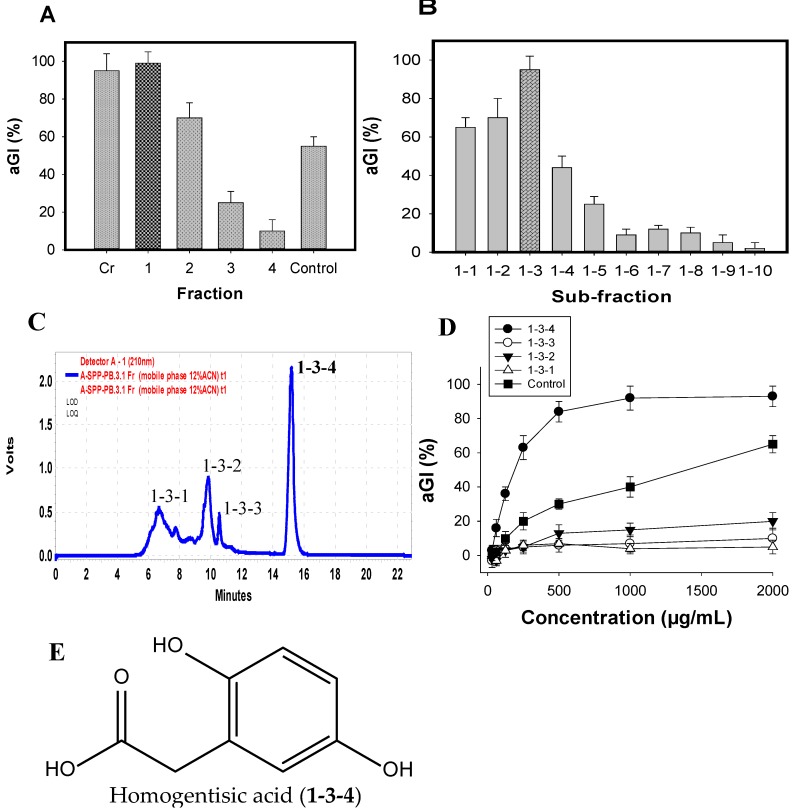
aGI %of fractions, sub-fractionation and compounds extracted from FSPP after Diaion open column (**A**); ODS open column (**B**); and Pre-HPLC column (**C**); respectively; the HPLC profile of sub-fraction1-3 (**D**); and the chemical structure of active compound (**E**).

The major active components were efficiently isolated by the application of various columns, including Diaoin, Octadecylsilane (ODS) open columns, and preparative high-performance liquid chromatography (Pre-HPLC), coupled with a biological-guided assay. The crude sample (FSPP) was primarily fractionated into four fractions (Fr.1–4) via a Diaoin open column. Fr.1 eluted with distilled water showed the strongest inhibition (99%) among these fractions, FSPP and positive control (acarbose) (Figure 4A); it was then chosen for further separation via an ODS open column with the MeOH/H_2_O gradient elution from 0/100 to 100/0. As shown in Figure 4B, sub-fraction1–3 possessed the most potential activity; thus, this sub-fraction was selected for further isolation of active compounds.

Four components (1-3-1, 1-3-2, 1-3-3, and 1-3-4) were isolated from sub-fraction1-3 (Figure 4C) by using Pre-HPLC with the mobile phase of 12% acetonitrile in H_2_O, and tested for their aGI activity (Figure 4D). Of these, 1-3-4 was found to be the most active inhibitor, showing the highest inhibition of 95%, which was higher than other components (0–20%) and positive control (acarbose, 66%). This target component was further separated for its greater purity by using the same Pre-HPLC column, with the modified mobile phase of 12% acetonitrile in 0.4% acetic acid, and recycling five times via the column, then tested activity. The chemical structure of the inhibitor was identified by using spectroscopic analyses including 1D (1H NMR, 13C NMR) and 2D NMR chemical shifts (1H-1H COSY, HSQC, and HMBC), coupling with the comparisons to those of previously reported compounds. The active compound (1-3-4) was identified as homogentisic acid (HGA) [36]. This isolated HGA possessed stronger activity than that of acarbose with the IC_50_ and maximum inhibition values of 220 μg/mL, 95%, and 1510 μg/mL, 65%, respectively. The commercial HGA compound was also tested; it showed the same activity as that of the isolated HGA. HGA was reported to show antioxidant and anti-inflammatory activities [35], and playing an important role in the metabolism of phenylalanine and tyrosine [37]. In this study, HGA was found to show aGI activity for the first time. Thus, HGA was characterized as a new inhibitor. Moreover, HGA does not contain sugar moieties (Figure 4E). In addition, non-sugar-based aGIs have received much attention in recent years due to their promising and efficient bioactivities [38]. Therefore, this new inhibitor could be a potential candidate for antidiabetic drugs.

## 3. Materials and Methods

### 3.1. Materials

Squid pens were purchased from Shin-Ma Frozen Food Co. (I-Lan County, Taiwan). *Paenibacillus* sp. TKU042 was obtained from the previous study [16]. Other bacterial strains, including: *Paenibacillus macerans* TKU029, *Paenibacillus mucilaginosus* TKU032, *Paenibacillus* sp. TKU037, *Bacillus* sp. TKU004, *Bacillus cereus*, *Bacillus mycoides* TKU038, and *Lactobacillus paracasei* subsp. *paracasei* TKU010, were stocked strains of the “Microorganisms and Biochemistry Laboratory” at the Department of Chemistry, Tamkang University, New Taipei City, Taiwan. Shin-Ma Frozen Food Co. (I-Lan County, Taiwan) provided the squid pens for this study. Rice α-glucosidase (Type 4) and porcine α-amylase (Type VI-B) were purchased from Sigma-Aldrich, St. Louis, MO, USA. Αlpha-amylase from *B. subtilis* was obtained from Challenge Bioproducts Co., Ltd., Yunlin, Taiwan. *Saccharomyces cerevisiae* (yeast), *Bacillus stearothermophilus* (bacterial) α-glucosidases and acarbose were obtained from Sigma Chemical Co., St. Louis, MO, USA. Rat intestinal acetone powders (rat α-glucosidase) were provided by Sigma-Aldrich, Singapore. *P-*nitrophenyl glucopyranoside (*p*NPG) was purchased from Sigma-Aldrich, St. Louis, MO, USA. ODS gel and Diaion HP-20 were acquired from Merck, and Mitsubishi Chemical Co., Tokyo City, Japan, respectively.

### 3.2. Enzymatic Inhibitory Assay

The enzymatic inhibitory assay was modified from the methods of Nguyen et al. (2017) [16]. Pre-incubation was started by mixing 50 µL of the sample and 50 µL of the enzymatic solution in a 100 µL potassium phosphate buffer. The mixture was then kept at 37 °C for 20 min to allow the inhibitors to combine with the enzymes. The reaction started when 50 µL of *p*NPG was added to the above mixture. This stage was maintained at 37 °C for 40 min when testing for inhibition against rat glucosidase, but only 30 min when yeast, rice, or bacterial α-glucosidases were used instead. The addition of 1 mol/L Na_2_CO_3_ solution (100 μL) stopped the reaction, and the optical density of the final solution was measured at OD_410nm_ (wavelength of 410 nm). The inhibitory activity was calculated using the following formula:

[**Inhibition** (%) = (**A** − **B**)/**A** × 100], (1)

where **A** is the optical density at OD_410nm_ of the reaction mixture without the sample (inhibitor), and **B** is the optical density at OD_410nm_ of the reaction mixture with the sample. The inhibition was also expressed as U/mL and as an IC_50_ value. One U (unit activity) was defined as the volume of sample that could inhibit 50% of enzymatic activity, while the IC_50_ value was defined as the concentration of sample that inhibits 50% of enzyme activity under assay conditions. 0.1 mol/L, pH 7 potassium phosphate buffer was used to prepare the samples, substrate, and enzyme solutions. The solution of rat α-glucosidase was prepared as described in detail in the previous report [16]. Rice, yeast, and bacterial glucosidases were tested at concentrations of 0.10, 0.25, and 1.0 U/mL, respectively. α-amylase inhibitory activity was determined as per the methods described by Nguyen et al. (2017) [39]. The differences between the mean values of inhibition (*p* < 0.01) were analyzed using SAS version 9.4, Statistical Analysis Software. 

### 3.3. Optimization of Culture Conditions for Maximal aGIs Productivity

#### 3.3.1. Effects of Cultivation Time and Supplementary Air on aGI Productivity

One hundred mL of medium (initial pH 6.85) containing 1% SPP, 0.1% K_2_HPO_4_ and 0.05% MgSO_4_·7H_2_O in a 250 mL-Erlenmeyer flask was fermented by *Paenibacillus* sp. TKU042 at 30 °C and a shaking speed of 150 rpm for 6 d. Fermentation was performed under two sets of conditions at the same time: no supplementary air, and supplementary air once/day where the bottom covers of the Erlenmeyer flasks were opened for 30 s in a sterile culture cabinet. The culture supernatants harvested daily were used to detect activity (after centrifugation at 4000 rpm for 20 min) and bacterial growth (after centrifugation at 500 rpm for 10 min).

#### 3.3.2. Effects of Additional Parameters on aGI Productivity

To achieve the greatest aGI productivity, additional parameters of cultivation were tested, including: culture temperature (25 °C, 30 °C, 34 °C and 37 °C), percentage of air head space in the 250 mL-Erlenmeyer flask (20%, 32%, 48%, 60% and 72%), SPP concentration (0.5%, 1%, 1.5% and 2%) and bacterial seed amount (0.5, 1, 2, and 4 mL of bacterial seed at OD_660nm_ = 0.35). The supernatants were harvested on days 3–4 and used for bio-assays, after centrifugation at 4000 rpm for 20 min.

### 3.4. Measurement of pH and Thermal Stability

The measurement of pH and thermal stability was as per the methods described in detail by Nguyen et al. (2017) [16,37].

### 3.5. Experimental Procedures of Isolation and Identification of the Major Inhibitor

Ten grams of FSPP were fractioned by a Diaion opened column (4 cm × 30 cm); the column was then successively washed with one liter of distilled water, 30% EtOH, 70% EtOH, and 100% EtOH to obtain four fractions (Fr.) of Fr. 1 (5.2 g), Fr. 2 (1.8 g), Fr. 3 (2 g), Fr. 4 (1 g), respectively. Fr. **1** (4g) was sub-fractionated by loading onto an ODS gel open column (5 cm × 6 cm), and then eluted with 0%, 10%, 20%, 30%, 40%, 50%, 60%, 70%, 85%, and 100% methanol provided to 10 sub-fractions (**1-1** to **1-10**, respectively). The potential sub-fraction **1**–**3** (eluted with 20% methanol) was further purified by Pre-HPLC [preparative Cosmosil 5C18-AR-II column; equipped with a 250 × 20 mm i.d. (Nacalai Tesque, Inc., Kyoto, Japan)] with the mobile phase of 12% acetonitrile, flow rate at 10 mL/min, detected at 210 nm, to result in isolation of a major active component **1**–**3**–**4**. This component was recycled five times with the use of the same Pre-HPLC column, and the modified mobile phase of 12% acetonitrile in 0.4% acetic acid. The active compound was identified by using NMR data including 1D NMR (1H NMR, 13C NMR) and 2D NMR (1H-1H COSY, HSQC, and HMBC) chemical shifts, coupled with the comparisons to those of previously reported compounds.

## 4. Conclusions

*Paenibacillus* species were used to convert squid pens, a fish processing by-product, to active aGIs. Among those tested, *Paenibacillus* sp. TKU042 was the most active aGI-producing strain; it was therefore chosen to determine the optimal culture conditions. aGI productivity increased 3.1-fold after the optimization process. The aGIs were strongly thermostable at 40–100 °C and could also retain high relative activity over a large pH range (2–13). The aGIs demonstrated efficient inhibition against α-glucosidases from rat, yeast, and bacteria, but weak inhibition against rice α-glucosidase. A major inhibitor was isolated from the fermented SPP and identified as homogentisic acid (HGA). HGA was found as a new inhibitor, non-sugar based aGI, and showed stronger activity than acarbose. The results suggest that SP is a viable C/N source for the production of active antidiabetic materials via bioconversion by *Paenibacillus*.

## Figures and Tables

**Table 1:** Comparison of aGIs induced by *Paenibacillus* and other bacterial species

No.	Bacterial Strain	Rat α-Glucosidase Inhibition		Yeast α-Glucosidase Inhibition	
		%	U/mL	%	U/mL
1	*Paenibacillus* sp. TKU042	83	335	98	560
2	*Paenibacillus* sp. TKU037	80	300	85	500
3	*Paenibacillus mucilaginosus* TKU032	76	298	79	450
4	*Paenibacillus macerans* TKU029	78	305	85	510
5	*Bacillus* sp. TKU004	-	-	-	-
6	*Bacillus cereus*	-	-	-	-
7	*Bacillus mycoides* TKU038	-	-	-	-
8	*Lactobacillus paracasei* subsp *paracasei* TKU010	-	-	-	-
Control (medium without bacteria)		-	-	-	-
Acarbose (commercial aGI)		88	ND	64	ND

The medium containing 1% SPP was fermented by the test bacteria. Fermentation processes were performed at 30 °C, 150 rpm shaking speed, with 100 mL of medium and 1 mL bacterial seed solution (OD660 nm = 0.35) over 3 d. The culture supernatants were centrifuged at 4000 rpm to remove medium residue and bacterial mass; the solution obtained was used for testing aGI. The activity was expressed as % and U/mL. (-): no activity; ND: not determined.

**Table 2:** Specific inhibitory activity of FSPP and acarbose

Enzyme	Inhibition of FSPP		Inhibition of Acarbose *	
	IC_50_ (µg/mL)	Maximum Inhibition (%)	IC_50_ (µg/mL)	Maximum Inhibition (%)
Yeast α-glucosidase	252 ± 16 ^c,d^	99 ± 1.2	1495 ± 170 ^a^	64 ± 3.5
Rat α-glucosidase	362 ± 13 ^c^	82 ± 3.3	117 ± 16 ^c,d,e^	88 ± 3.4
*Bacterial* α-glucosidase	189 ± 17 ^c,d,e^	85 ± 2.3	0.015 ± 0.001 ^e^	100 ± 2.2
Rice α-glucosidase	773 ± 59 ^b^	60 ± 4.5	3.89 ± 0.9 ^d,e^	100 ± 1.9
Porcine pancreatic α-amylase	-	-	ND	ND
*B. subtilis* α-amylase	-	-	ND	ND

(-): No inhibitory activity; ND: not tested; CV = 25.98193; LSD_0.01_ = 251.97; triplicates of each experiment *(n* = 3). IC_50_ values with the same letters are not significantly different based on *t*-test ranking.
